# How COVID‐19 shifted the seasonal flu in Korea

**DOI:** 10.1111/irv.13113

**Published:** 2023-03-01

**Authors:** Seunghyun Lewis Kwon, Bryan Inho Kim

**Affiliations:** ^1^ Division of Immunization Korea Disease Control and Prevention Agency Cheongju Republic of Korea; ^2^ KDI School of Public Policy and Management Sejong Republic of Korea; ^3^ Division of Infectious Disease Control Korea Disease Control and Prevention Agency Cheongju Republic of Korea

**Keywords:** COVID‐19, influenza, influenza‐like illness, Republic of Korea, vaccination

## PEER REVIEW

The peer review history for this article is available at https://publons.com/publon/10.1111/irv.13113.

**FIGURE 1:**
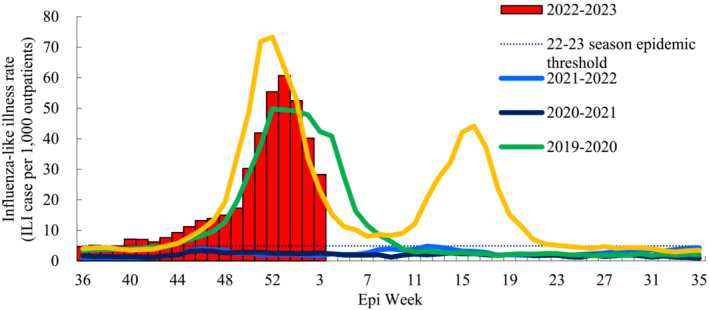
Weekly influenza‐like illness rate in the Republic of Korea, 2018–2019 to 2022–2023.

Prior to 2021, Republic of Korea had experienced typical seasonal epidemics of influenza, with some seasons showing higher rates of influenza‐like illness. Typically, seasonal flu epidemics in the country are reported between December and March.^1^ In the 2019–2020 season, there was a relatively high number of ILI rates. Notably, during the 2020–2021 and 2021–2022 seasons, the ILI rates had remained below the epidemic threshold, as Figure 1 illustrates. There had been continuous concern for the possible “twindemic” since the emergence of COVID‐19. In the 2021–2022 season, this concern had grown with the ease of non‐pharmaceutical interventions, including social distancing measures. “Twindemic” is a term used to describe the simultaneous occurrence of two different epidemics: COVID‐19 and the seasonal influenza. It is used to stress the possible subsequent burden on healthcare systems and on the overall society.

The 2022–2023 flu season in the Republic of Korea has shown a similar pattern to seasons prior to the COVID‐19 pandemic. Despite an increase in the ILI baseline since Week 40, no early increase was observed. ILI rates increased above the seasonal threshold (4.9 ILI cases per 1000 outpatients) in Week 40, gradually increasing until Week 49, and showing a typical exponential increase starting from Week 50, similar to the pattern before the COVID‐19 pandemic. The peak was observed in Week 53 and has been decreasing since then. This reoccurrence of the typical flu epidemic in the Republic of Korea is not surprising, given the lifting of overall social distancing measures and reduced population immunity to seasonal influenza due to the absence of a flu epidemic in the 2020–2021 and 2021–2022 seasons.^2^ Influenza vaccination rates have been stable during the COVID‐19 pandemic.^3^ Nevertheless, the indoor mask mandate has been maintained, which may have prevented an early surge of the seasonal flu epidemic, unlike in other countries, including Australia and the United States.^4^, ^5^, ^6^

The impact of the resurgence of the seasonal flu in the midst of the Omicron variant‐driven COVID‐19 pandemic has not been as catastrophic as feared in the country. There is a chance for a spring season second peak since the indoor mask mandate is set to be lifted by the end of January 2023. Therefore, close monitoring of seasonal flu and COVID‐19 trends, as well as continuous encouragement of vaccination for both highly transmissible pathogens, is needed. At present, the worst‐case scenario appears to have been averted. Nevertheless, it is imperative that we continue to exercise caution, as it is currently too early to lower our guard.

## AUTHOR CONTRIBUTIONS

**Seunghyun Lewis Kwon:** Conceptualization; formal analysis; methodology; writing – original draft; writing – review and editing. **Bryan Inho Kim:** Methodology; supervision; validation; writing – original draft; writing – review and editing.

## CONFLICT OF INTEREST STATEMENT

The authors declare that they have no known competing financial interests or personal relationships that could have appeared to influence the work reported in this paper.

## Data Availability

These data were derived from the following resources available in the public domain: https://www.kdca.go.kr/board/board.es?mid=a30504000000&bid=0033.

## References

[1] Park JY , Kim HI , Kim JH , et al. Changes in respiratory virus infection trends during the COVID‐19 pandemic in South Korea: the effectiveness of public health measures. Korean J Intern Med. 2021;36(5):1157‐1168. doi:10.3904/kjim.2021.026 34399570 PMC8435496

[2] Dhanasekaran V , Sullivan S , Edwards KM , et al. Human seasonal influenza under COVID‐19 and the potential consequences of influenza lineage elimination. Nat Commun. 2022;13(1):1721. doi:10.1038/s41467-022-29402-5 35361789 PMC8971476

[3] Park H , Kwon SL , Park J , et al. Immunization program against influenza in the Republic of Korea 2021‐2022 season. Public Health Wkly Rep. 2022;15(26):1835‐1849.

[4] Trent MJ , Moa A , MacIntyre CR . “I'll be back”: Australia's experience of flu in 2022. BMJ. 2022;379:o2998. doi:10.1136/bmj.o2998 36517068

[5] Thomas CM , White EB , Kojima N , et al. Early and increased influenza activity among children—Tennessee, 2022–23 influenza season. MMWR Morb Mortal Wkly Rep. 2023;72(3):49‐54. doi:10.15585/mmwr.mm7203a1 36656786 PMC9869745

[6] Kuitunen I , Renko M , Tapiainen T . Unusual late epidemic peak during influenza season 2021‐2022: a nationwide register‐based analysis in Finland. Influenza Other Respi Viruses. 2022;16(6):1199‐1201. doi:10.1111/irv.13036 PMC9530521 36047516

